# Intra-Uterine Peritonitis in the Fœtus

**Published:** 1849-01

**Authors:** 


*Intra-Uterine Peritonitis in the Fœtus.*—Dr. Simpson exhibited at the meeting of the Edinburgh Obstetrical Society a fœtus, which had evidently laboured under peritonitis, as was evidenced by the effusion of lymph on various parts of the peritoneum. He believed that the disease was more frequent than is suspected, as he had discovered its traces in many fœtuses which had died in the seventh or eighth month of utero-gestation.—*Monthly Journal.*

